# Supplementary figures and images for: Longitudinal bioluminescent imaging of HIV-1 infection during antiretroviral therapy and treatment interruption in humanized mice
Source: PLoS Pathog. 2019 Dec 5;15(12):e1008161. doi: 10.1371/journal.ppat.1008161 (PMC6917343; doi:10.1371/journal.ppat.1008161)

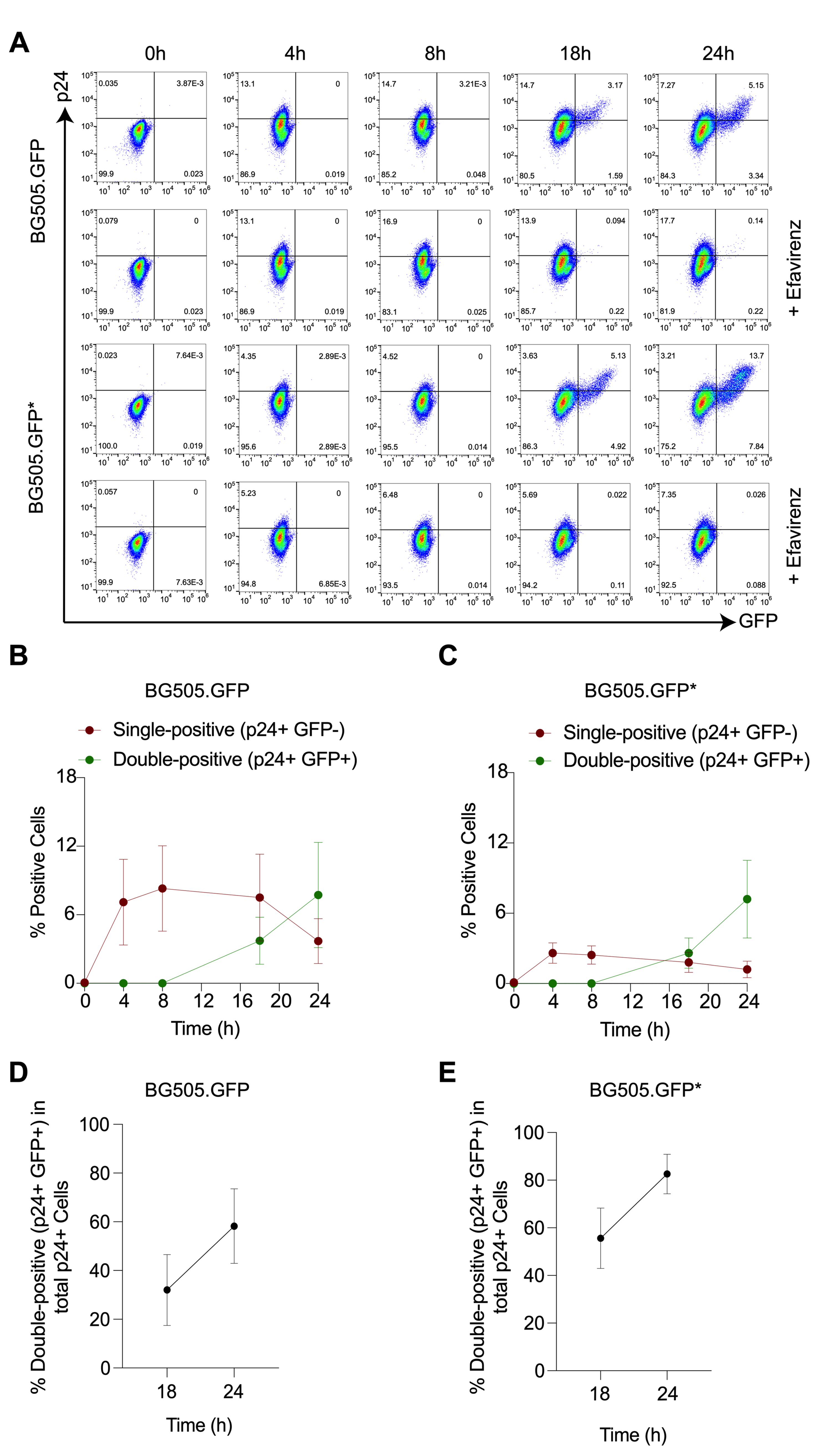

Supplement: S1 Fig — (A) GFP reporter gene expression at different timespoints during the initial 24 hours of infection in CEMSS.NKr.R5 cells with or without the addition of 1 μM Efavirenz. Data is representative of three independent experiments. (B-C) Percent of single positive (p24+ GFP-) and double-positive (p24+ GFP+) cells over 24 hours in CEMSS.NKr.R5 cells infected with (B) BG505.GFP or (C) BG505.GFP* T/F reporter viruses. Data displayed as the mean +/- SEM of three independent experiments (n = 3). (D-E) The fraction of double-positive cells in the total HIV-1 infected (p24+) population 18 hours and 24 hours post-infection in CEMSS.NKr.R5 cells infected with (D) BG505.GFP or (E) BG505.GFP* T/F reporter viruses. (TIF) [file ppat.1008161.s001.tif]

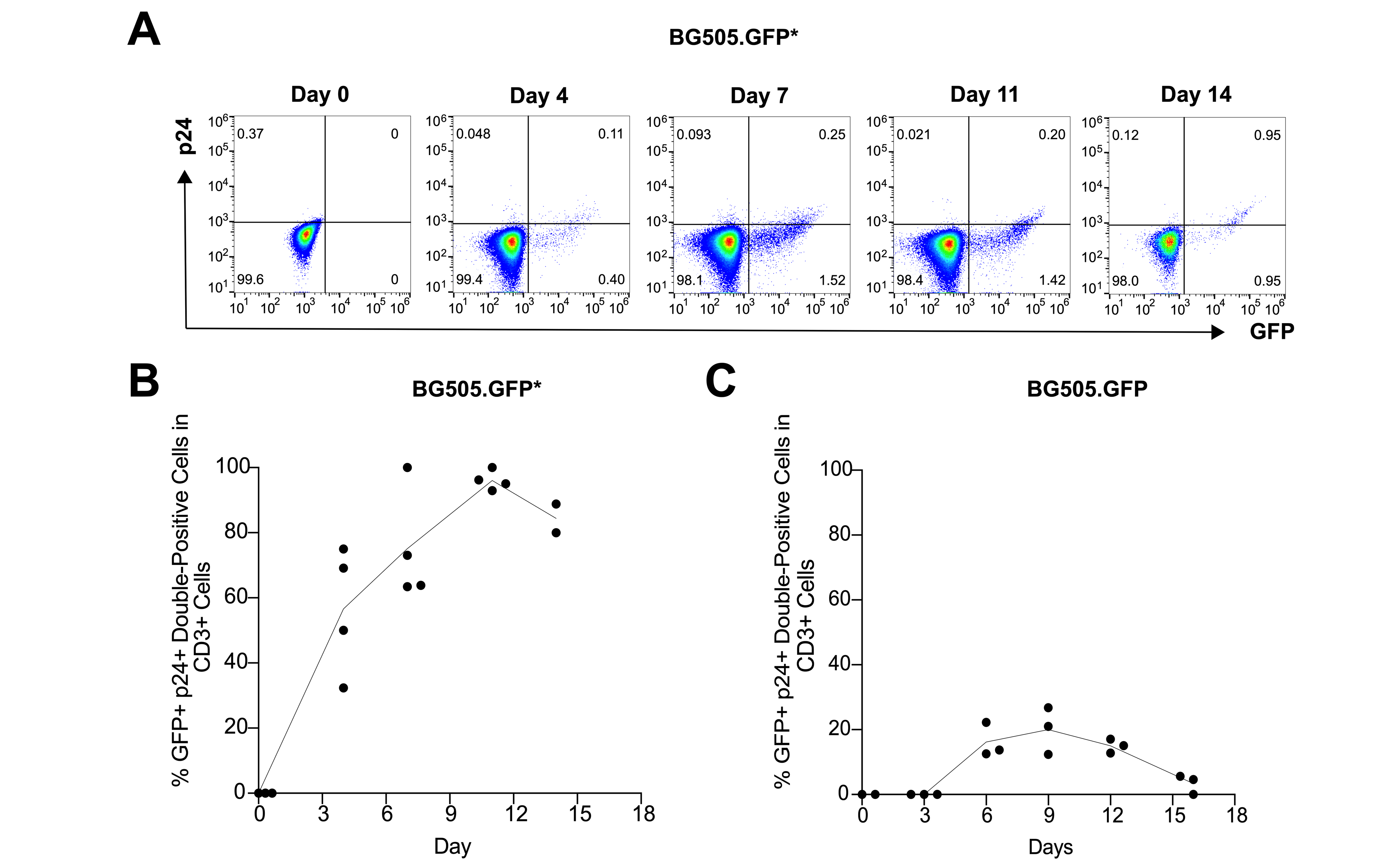

Supplement: S2 Fig — (A-C) GFP reporter gene stability in p24+ CD3+ PBMCs extracted from the peripheral blood of Hu-PBL mice infected intraperitonially (i.p.) with 1 x 10^7^ infectious units (IUs) HIV-1 T/F reporter virus. (A) Representative FACS plots gated on CD3+ PBMCs extracted from BG505.GFP* infected Hu-PBL mice (n = 4). Data is representative of four individual Hu-PBL mice. (B-C) Average GFP reporter gene stability in Hu-PBL mice infected with 1 x 10^7^ infectious units (IUs) of BG505.GFP* (n = 4) (B), and BG505.GFP (n = 3) (C) T/F reporter virus for 14–16 days. Data displayed as the percentage of GFP and p24 double-positive cells in the total p24+ population. A line crosses the average percent GFP expressing cells within the total p24+ cell population for mice analyzed at each time point. (TIF) [file ppat.1008161.s002.tif]

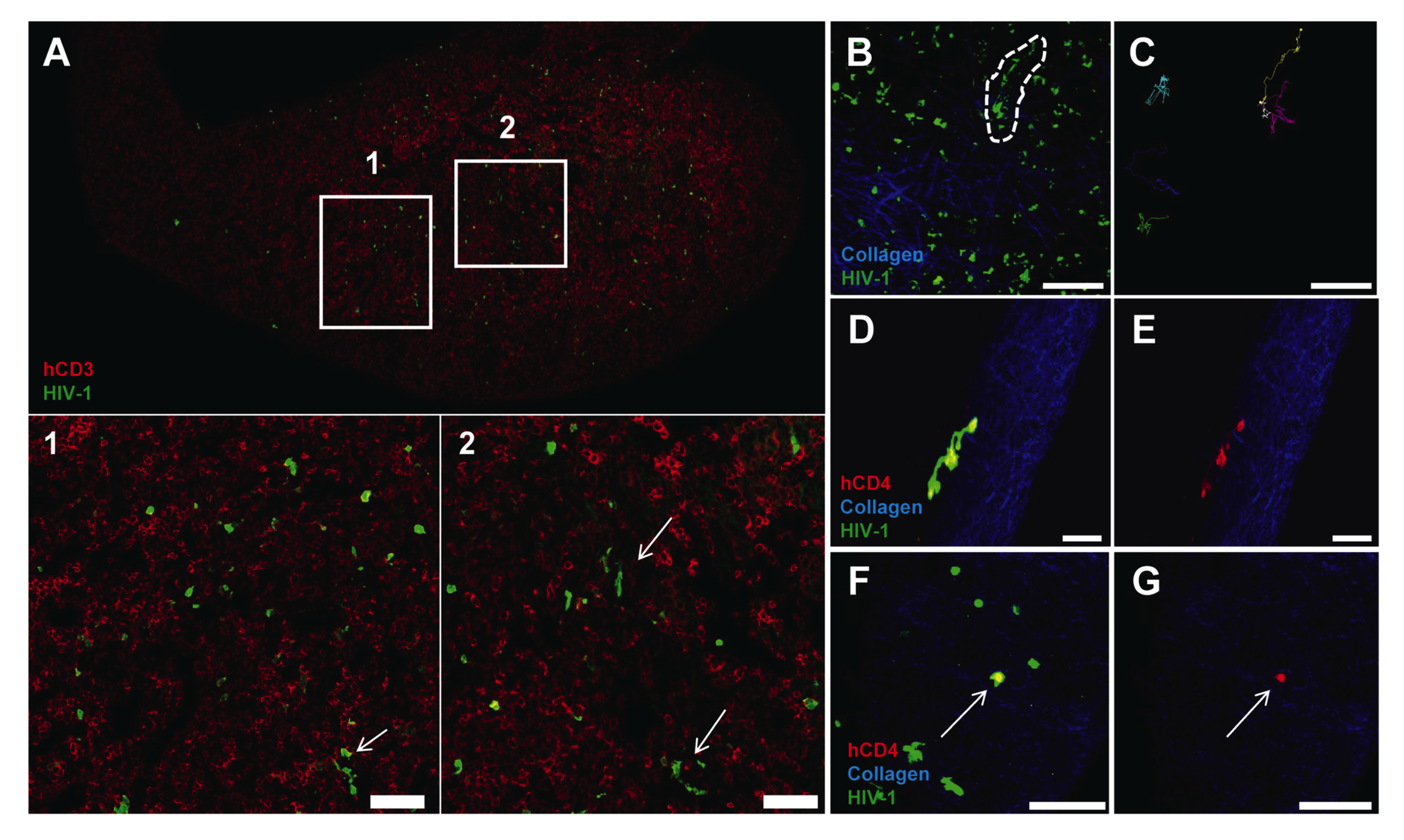

Supplement: S3 Fig — (A) Cryoimmunofluorescent confocal imaging of splenic tissue sections; areas with GFP expressing cells are magnified in panels 1 and 2. White arrows indicate putative syncytia formed during infection. (B-G) LS-MPM imaging of spleen tissue from a Hu-PBL mouse injected i.p. with 1 x 10^7^ IUs TRJO.GFP 7 days post-infection and injected with RFP expressing CD4 T cells 24 hours prior to imaging. (B,C) LS-MPM intravital imaging of an area in the spleen with GFP expressing cells. A representative cell exhibiting long membrane extensions is outlined in white dashes (B) with motion tracks of GFP expressing cells in (C). (D-E) LS-MPM image of GFP and CD4 co-expressing syncytium in the spleen of a TRJO.GFP-infected Hu-PBL mouse (D) and the same image with CD4 expression alone (E). (F-G) LS-MPM image of GFP expressing cells in the spleen as in (D) with a GFP and CD4 co-expressing cell indicated by the white arrow and CD4 expressing cells alone (G). All scale bars correspond to 100 μm. (TIF) [file ppat.1008161.s003.tif]

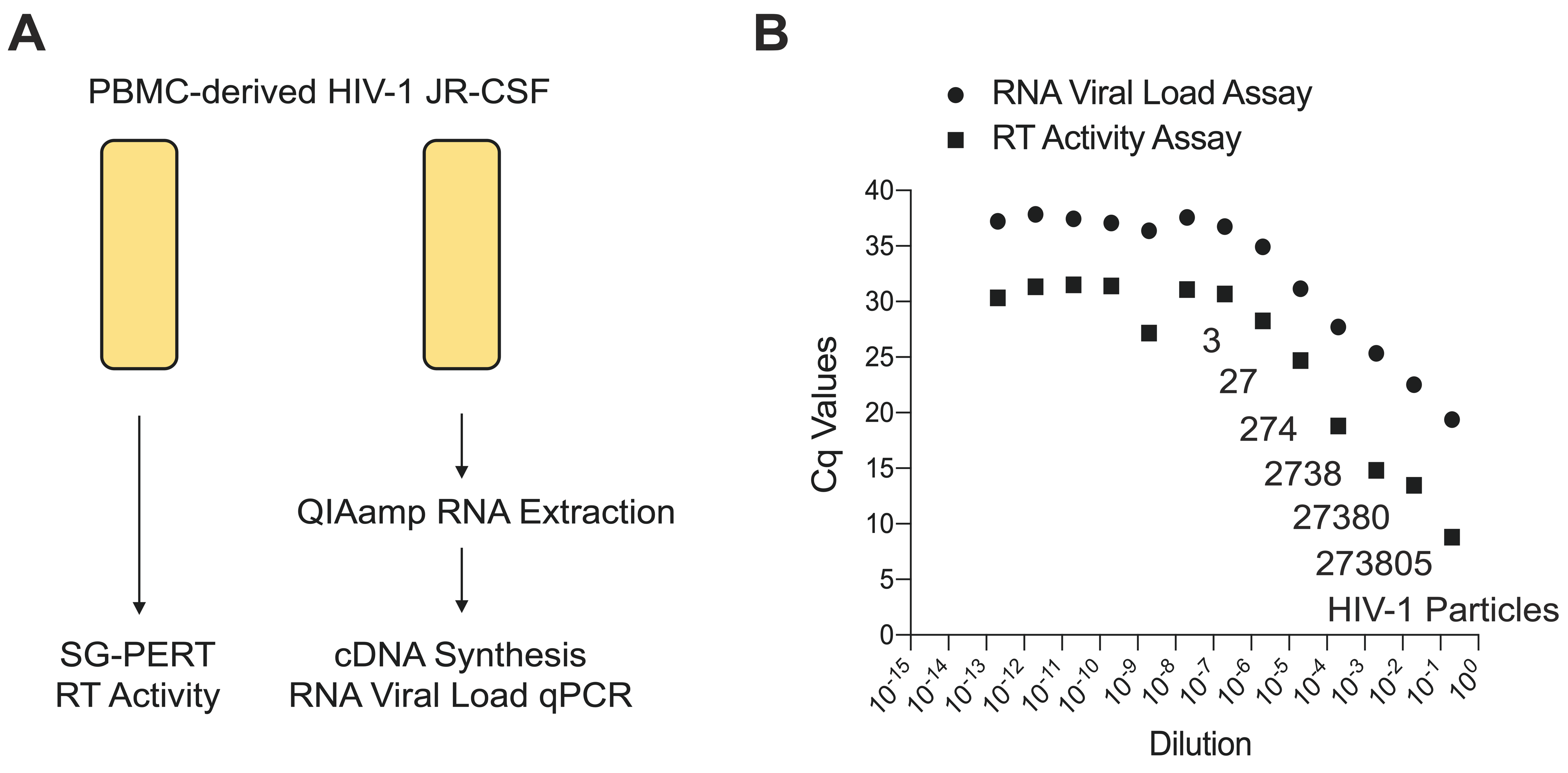

Supplement: S4 Fig — (A) Peripheral blood mononuclear cell (PBMC) derived HIV-1 JR-CSF viral supernatant was stored in separate aliquots of equal volume in order to compare the sensitivity of the Quantitect qRT-PCR viral load assay and the SG-PERT reverse transcriptase activity qPCR assay in parallel. (B) The Quantitect qRT-PCR viral load assay and the SG-PERT reverse transcriptase activity qPCR assay was run in parallel with viral RNA eluate and HIV-1 supernatant serially diluted until the limit of detection for each assay was reached. Data shown as the average cycle threshold (Cq) values determined from two technical replicates at each dilution. The limit of detection was defined as the Cq value at which the linear range of the assay ended. Absolute quantification of HIV-1 particles was determined from a viral RNA standard curve run in parallel with the Quantitect qRT-PCR viral load assay. (TIF) [file ppat.1008161.s004.tif]

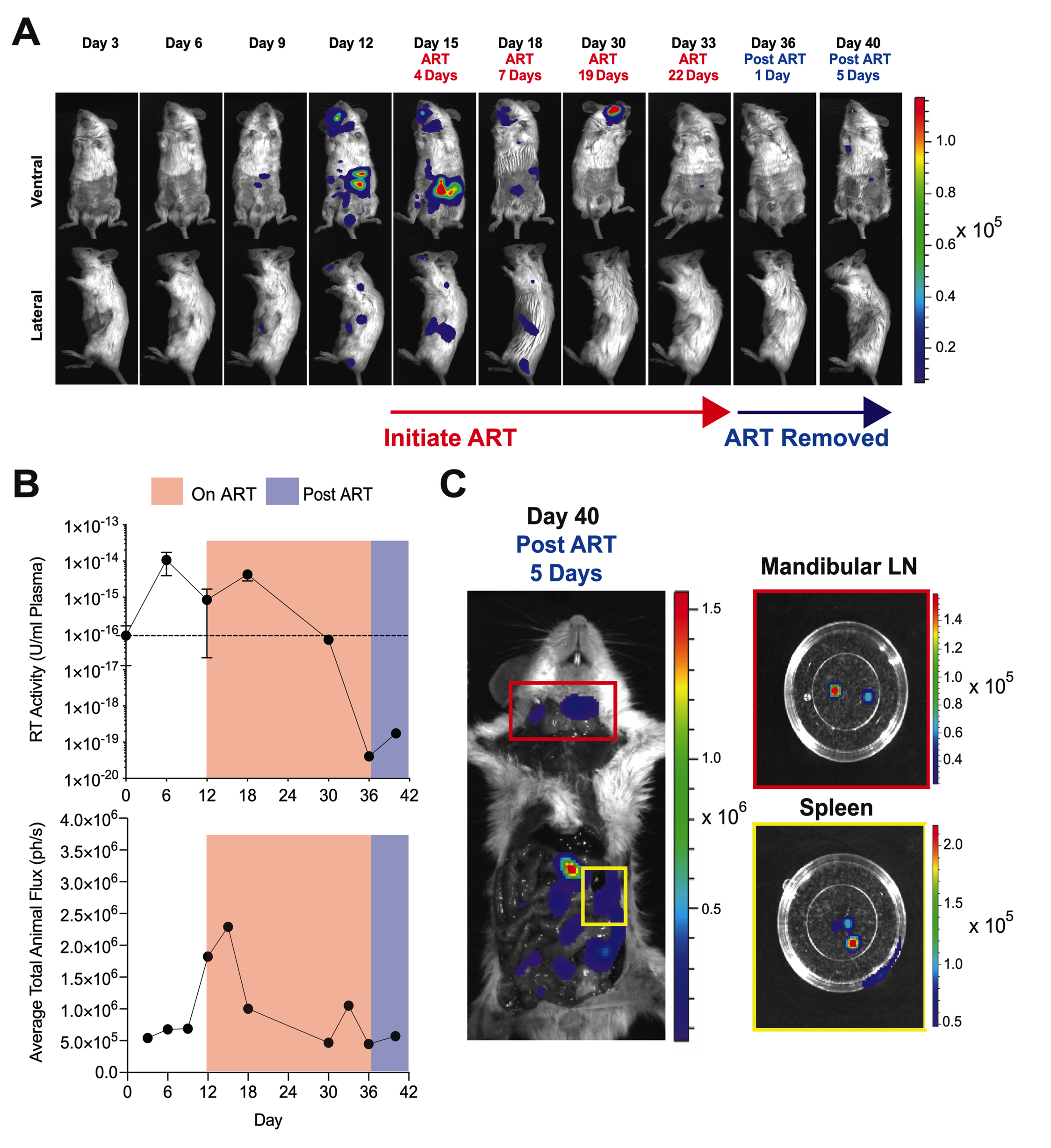

Supplement: S5 Fig — (A) Bioluminescent imaging of spreading infection of Hu-BLT Mouse #3 infected with 1 x 10^6^ IUs of Q23.BG505.Nluc T/F reporter virus and placed on a daily cART regimen comprised of daily i.p. cART injections of Truvada and Isentress 12 days post-infection. (B) Plasma reverse transcriptase activity from Hu-BLT Mouse #3 (above) and whole-animal Nluc signal (below) over the course of the 40-day imaging period. Plasma reverse transcriptase activity in serum samples taken every six days over the course of the imaging period was measured via the SG-PERT reverse transcriptase activity assay and described as reverse transcriptase activity units / mL above endogenous uninfected background levels (dotted line). (C) Whole animal ex vivo necroscopic analysis of rebounding infection in Hu-BLT Mouse #3 five days following cART cessation. (TIF) [file ppat.1008161.s005.tif]
